# Pure Milk

**Published:** 1897-05

**Authors:** R. A. Pearson

**Affiliations:** Assistant Chief, Dairy Division, United States Department of Agriculture


### PURE MILK.

By R. A. Pearson, B.S.,
ASSISTANT CHIEF, DAIRY DIVISION, UNITED STATES DEPARTMENT OF AGRICULTURE.

(Concluded from page 193.)

The chief sources of infection are dirt and water. Dirt finds entrance to the milk in many ways; perhaps most frequently it falls into the pail while the milk is being drawn. Every time the udder is jarred by the hand, or the hair on the thigh and side is disturbed by the arm or the motion necessary in milking, particles of dirt, epithelial cells, and hairs are loosened, and many of them fall within the pail, bringing with them myriads of germs of numerous forms. When animals remain in the stable all the time, or are confined to the ordinary barnyard, they become covered with loose particles of dirt, and, if they are not cleaned, it is not uncommon to find a large amount of dirt in the bottom of the milk-vessel after the milk has stood undisturbed a short time.

This source of infection may be easily overcome. Farmers often think that the horse must be well cleaned, but it makes little difference with the cow; this is a great mistake. Cows should be groomed daily, and before they are milked the udder and surrounding parts should be carefully cleaned and wiped with a damp cloth, so that the particles of dirt and bacteria that remain will adhere to the animal and not be so easily shaken off, as is the case when they are dry. It is also important for the milker to be clean. Hands should be carefully washed and overclothes used for no other purpose should be put on before milking is commenced. At the Experiment Station in Wisconsin the effect of cleanliness on the part of the cow and milker has been shown. It was found that under ordinary conditions the number of bacteria deposited per minute in an ordinary-sized milk-pail during milking was over 16,000, while this number was reduced to about 2500 when the precautions just described were taken, a reduction of 85 per cent. Many similar experiments prove the importance of having cow and milker both clean.

Improperly cleaned dairy utensils are another prominent source of milk-infection. Old, worn, battered pails and cans should be discarded, as they cannot be thoroughly cleaned, and those used for carrying milk should be used for no other purpose, nor should they be allowed to remain in the stable when not required there. Vessels are frequently washed by agitating water in one and pouring it into another, continuing this even after it becomes cool and unfit for use. They should be carefully cleaned in hot water, rinsed in clean water, and sterilized with live steam or boiling water. Russell has found that milk taken in sterile pails contained but 165 germs per c.c., while that in unsterilized pails taken under the same conditions contained over 4000 germs per c.c. The sterilization of milk-utensils should be universally practised; it takes away danger of infection by pathogenic and non-pathogenic bacteria that may remain after washing or have been introduced by the rinse-water.

Germ-laden air is responsible for many bacteria obtaining entrance to the milk. When hay or any other dry fodder is fed much dust arises from it, and the number of germs falling within a space the size of the opening of an ordinary milk-pail may be over 150,000 per minute. It is, therefore, important to use such feed-stuffs *after* milking, or to moisten them if they are to be fed just previous to or during milking. The air of cities is often teeming with micro-organisms, and it is liable to contain pathogenic bacteria. For this reason, if for no other, it is essential that dairies which are to produce pure milk should be located in the country.

The fore-milk is a source of infection not to be overlooked. A little milk usually remains in the teat, and this is easily reached by some of the bacteria from the outside of the udder; all the conditions are favorable to their growth, and they multiply with astonishing rapidity. Usually the germs in the teat represent few species, but the number of individuals is very great, and they may infect all that is drawn into the same vessel as the fore-milk. Shultz has found almost 100,000 germs per c.c. of fore-milk.

It was found that milk as ordinarily taken contained over 15,000 bacteria per c.c., while with the precaution above suggested there were less than 350 in the same volume, or a diminution of about 98 per cent. Thus it is seen that the number of germs entering milk can be largely controlled by observing cleanliness in every detail of the work.

The rapid cooling of milk is of equal importance. After it has been drawn it should be immediately cooled to 45° F., or lower, to check bacterial growth. If this is not done, it will soon contain as many organisms as it would under ordinary circumstances, and the extra care that has been exercised will be wasted. Each pailful should be cooled as it is emptied, and not allowed to remain warm even ten or fifteen minutes until a can is filled. In the best-conducted dairies special stress is placed on the cooling of the milk; in some cases it is at a temperature of 40° within fifteen minutes after it has been drawn from the cow. Spores require a higher temperature for germination than is necessary for the growth and multiplication of germs. So if milk is cooled immediately, it will be quickly brought to a temperature at which the spores falling into it will not germinate; but if cooling is slow, many spores will have ample time to germinate while the milk is still warm and rapidly reproduce at the lower temperature at which they would not have been affected.

Non-pathogenic germs have been referred to chiefly; but if the germs of a contagious disease are in the neighborhood of a dairy, they may be conveyed to the milk exactly in the same way as the harmless ones. The cow herself may be a source of contamination. If she is diseased, germs may be found in the milk in the udder. Germs of tuberculosis are transmitted from the animal in this way, also those of diphtheria, scarlet fever, and foot- and mouth-disease. Guillebeau conducted a series of experiments in which he examined the milk of seventy-six cows suffering with inflammation of the udder, and in each case he found the milk to contain bacteria which produced, by inoculation, similar trouble in healthy animals. The bacillus of tuberculosis is probably the pathogenic organism now most to be feared in milk; this insidious disease has attracted much attention from physicians, veterinarians, and lawmakers. The number of cattle infected with it is variously estimated at from 2 to 10 per cent. Bang and Ernst have shown that cows having general tuberculosis, but no disease of the udder, may produce milk infected with the bacillus tuberculosis, and other authorities concur with them. All agree that it is not safe to use the milk from cows whose udders are affected by tubercular disease. The physical examination for tuberculosis is not reliable, and it is difficult to detect the presence of these bacteria by microscopic examination, and inoculation experiments are slow. Many large herds may be found that have been examined with the aid of the tuberculin-test, which is now considered the best proof of the existence or non-existence of tuberculosis. The bacilli of tuberculosis are not as easily killed as those of most other diseases. They are not affected in the alimentary canal, and a temperature of 150° for about an hour or 160° for fifteen minutes seems to be necessary to kill them.

Other sources from which pathogenic bacteria are derived are sick persons who attend the animals or have care of the milk. Typhoid fever is, perhaps, the most prominent of these diseases, and quite a number of outbreaks supposed to have been caused by infected milk are recorded. Busey and Koben report the circumstances of an outbreak at Stamford, Conn. In 1893 there was an outbreak of thirty cases of scarlatina in Glasgow, and it is described by Busey and Koben as follows: "This epidemic was traced to the milk from two dairy farms. At one of these a boy simply suffered from sore-throat which induced scarlet fever in others. At the other farm some of the dairy employés developed scarlet fever and had kept at work for one or two days after illness." Twenty-eight cases of diphtheria occurring at Hightstown, N. J., are referred to as follows: "These cases occurred within one week and were all traced to a milk-supply derived from a farm where a German boy assisted in milking while he had diphtheria."

Although it is true that the common bacteria of milk are not pathogenic, some of them by their growth may produce poisonous toxins, and when they are present in large numbers serious results upon the consumer may be produced by these products. Cholera infantum and other intestinal troubles are often attributed to this cause.

With all these facts before us, we would say that the following are the chief things to be observed in establishing a dairy plant:

Any animal suffering from any disease, or not in a normal condition, should be excluded from the herd.

The pastures should be free as possible from foul, decaying animal or vegetable matter. The cows should not have access to swampy ground or any place where they may become unnecessarily befouled.

The stables should be constructed with a view to the comfort and health of the stock and the convenience of the employés. They should be on elevated, well-drained ground, and lighted and ventilated in the most approved manner. The waste-products should be frequently removed, and the feed should be kept apart from the stock-room. No waste-products should be allowed to accumulate in the vicinity of the building.

The water-supply should be abundant and pure, and obtained from deep wells when possible. Springs, brooks, and shallow wells may easily be contaminated, and water from them should be used with caution.

No unwholesome foods should be used, nor any that has begun to putrefy or spoil. Any feeding materials that might impart an objectionable taste or flavor to the milk should be avoided.

The dairy-house should be located and constructed with the same care as the stable. The drainage needs special attention.

The animals should be sheltered at all times when the weather might be prejudicial to their health. They should be cleaned every day, and made comfortable at all times. Kind treatment is imperative, and the milk from any animal in an excited condition should not be used.

Milking should take place in a clean, well-ventilated building, and the milk should be drawn in a cleanly manner by tidy attendants. The vessels should be clean and sterilized. The milk should be strained through fine-wire mesh and cloth and cooled, and held in a place free from contamination at a temperature not exceeding 50° F. It should be served in glass jars which have been thoroughly cleaned and sterilized.

It has been suggested that the place where the improvement of the milk-supply should begin is on the farm, and improvement should be made at each step from the farm to the consumer. Intentional fraud and negligence are less to be feared than ignorance, hence education is one of the chief remedies to be sought. A good start toward educating dairymen has already been made by the establishment of dairy-schools, one being located in every State where dairying is a prominent industry. Courses are given at these schools in the winter and continued from six to twelve weeks in all, all the time being devoted to the study of dairying and closely allied subjects, such as the selection, care, and management of the dairy herd, the composition of milk and its products, the manufacture of butter and cheese, dairy chemistry and dairy bacteriology. Young men who have taken these courses are now connected with firms supplying milk to cities, and this special training has proved to be of much value to them and to their employers.

Dairymen and others will gradually learn to use greater intelligence in the production and handling of milk. They will learn to cover their field more perfectly than most of them do at present, and the veterinarian will be called upon more frequently for assistance.

The consideration of pure milk and aid in obtaining it seems to fall naturally to veterinarians. They are concerned with animals and their products. On account of their profession they understand the functions by which milk is produced and know something of its nature; being interested in the general health of a community, they should assist in having pure foods. And from a business standpoint it is to their interest to have the milk-consumption as large as possible.

As suggested above, one of the chief agents which has acted to increase milk-consumption has been the increased confidence of consumers. What can better serve this end than for them to know that the dairy, the stock, the surroundings, and the methods of conducting the work are carefully and frequently examined by a competent veterinarian who has made milk a special study? A veterinarian is already qualified for this work, though it may be advisable for him to brush up a little on the question of bacteriology, with special reference to the dairy.

Special attention should be given to prevent the entrance of pathogenic germs to the milk, and frequent examinations of the animals, employés, and surroundings of the dairy should be made. An examination of the herd in the field is not sufficient—every individual cow should be examined; the health of the employés and their families should be ascertained, and other things requiring attention, and feed, water-supply, the conditions of the stable and its ventilation, the milk-room, milk utensils, and methods of handling milk.

A good start toward supplying our cities with sanitary milk has been made. In almost all large towns it is possible to obtain milk that is produced on scientific principles. But places that can be thoroughly relied upon are yet few. They have many obstacles to overcome; one of them is the ignorance of many persons as to the value of pure milk. Some form of dairy inspection whereby the public may have full knowledge of the conditions under which the milk it consumes is produced is recommended. Various methods are followed in European countries and in the States. It is believed that one of the best systems has been inaugurated in New Jersey, and the milk from the dairy in question is termed "certified," as a body of experts certify that it is pure. A number of physicians interested in improving the milk-supply of their city have drawn up a contract with a dairyman which provides for the proper conduct of every part of the business; it specifically states the points upon which special importance is placed relating in detail to the duties of the dairyman.

The following section from the proposed contract is the one relating to the surroundings: "It is further understood and agreed that the immediate surroundings of the building shall be kept in a condition of cleanliness and in order. There shall not be allowed to accumulate in the vicinity any loose dirt, rubbish, or decayed vegetable or animal matter or animal waste, nor shall there be within three hundred yards of any building any constantly wet or marshy ground or stagnant pools of water; nor shall there be kept within three hundred yards of any building used for dairy purposes any fowls, hogs, horses, or other live-stock."

The physicians of the commission serve without compensation; they employ a veterinarian, a bacteriologist, and a chemist to examine the dairy and milk at certain intervals, and the reports of these experts are printed, all expenses being paid by the dairyman. As samples of milk for examination are liable to be taken from the wagon at any time, the reputation of the dairyman is constantly at stake. Milk produced under these conditions and placed on the market with the recommendation which it carries commands an extra price, which is willingly paid by those who appreciate its value, and is necessary on account of the increased cost of production.

Whether or not the plan could be successfully followed in other cities is uncertain. In the place referred to there had been an outbreak of typhoid fever which was traced to the milk-supply, and physicians and all citizens were alike aroused to the importance of radical reforms. In many cases a modification of the plan might be adopted; the Board of Health, or a committee appointed by them, might take the place of the medical board, and milkmen desirous of receiving the indorsement of this body should conform to their regulations and pay all expenses of the experts, who would make inspections and reports as often as requested by the board. Such a system could be conducted without expense to the community, and, being voluntary on the part of the dairyman, those who go into it should receive full benefit of it, such as privilege to make use of the experts' reports. It would be necessary to be very strict in regard to the conditions imposed and failure to comply with them.

There are about 17,000,000 cows in this country. It is estimated that 5,000,000 of them, whose annual product is valued at $150,000,000, produce milk for direct consumption. The task of improving this tremendous product is very great, and as there seems to be no one better qualified to assist the dairyman in the work than the veterinarian, it is most appropriate for the subject to come up for discussion in your meeting. I earnestly hope that greater interest may be awakened and some good accomplished by to-night's discussion.

